# Altered Expression of High Molecular Weight Heat Shock Proteins after OCT4B1 Suppression in Human Tumor Cell Lines 

**DOI:** 10.22074/cellj.2016.3832

**Published:** 2016-01-17

**Authors:** Mohammad Reza Mirzaei, Mohammad Kazemi Arababadi, Malek Hossein Asadi, Seyed Javad Mowla

**Affiliations:** 1 Department of Molecular Genetics, Faculty of Biological Sciences, Tarbiat Modares University, Tehran, Iran; 2 Molecular Medicine Research Center, Rafsanjan University of Medical Sciences, Rafsanjan, Iran; 3 Immunology of Infectious Diseases Research Center, Rafsanjan University of Medical Sciences, Rafsanjan, Iran; 4 Departments of Biotechnology, Institute of Science and High Technology and Environmental Sciences, Graduate University of Advanced Technology, Kerman, Iran

**Keywords:** HSPs, siRNA, Tumor Cell Lines

## Abstract

**Objective:**

OCT4B1, a novel variant of OCT4, is expressed in cancer cell lines and tis- sues. Based on our previous reports, OCT4B1 appears to have a crucial role in regulating apoptosis as well as stress response [heat shock proteins (HSPs)] pathways. The aim of the present study was to determine the effects of OCT4B1 silencing on the expression of high molecular weight HSPs in three different human tumor cell lines.

**Materials and Methods:**

In this experimental study, OCT4B1 expression was suppressed in AGS (gastric adenocarcinoma), 5637 (bladder tumor) and U-87MG (brain tumor) cell lines using RNAi strategy. Real-time polymerase chain reaction (PCR) array was em- ployed for expression level analysis and the fold changes were calculated using RT2 Pro- filer PCR array data analysis software version 3.5.

**Results:**

Our data revealed up-regulation of *HSPD1* (from *HSP60* family) as well as *HSPA14*, *HSPA1L*, *HSPA4*, *HSPA5* and *HSPA8* (from *HSP70* family) following OCT4B1 knock-down in all three cell lines. In contrast, the expression of *HSP90AA1* and *HSP90AB1* (from *HSP90* family) as well as *HSPA1B* and *HSPA6* (from *HSP70* family) was down-regulated under similar conditions. Other stress-related genes showed varying ex- pression pattern in the examined tumor cell lines.

**Conclusion:**

Our data suggest a direct or indirect correlation between the expression of OCT4B1 and HSP90 gene family. However, OCT4B1 expression was not strongly corre- lated with the expression of HSP70 and HSP60 gene families.

## Introduction

Based on the cancer stem cell hypothesis, adult stem cells (present in nearly all human tissues) or reprogrammed somatic cells are the primary source for tumor initiation (1,3). According to this hypothesis, the embryonic stem cell (ESC)-specific markers are re-expressed in tumor tissues and could therefore be employed as tumor markers for diagnosis or targeted therapy of various cancers. 

Octamer-binding transcription factor 4 (OCT4) belongs to a family of transcription regulatory proteins containing the POU DNA binding domain (4). *OCT4* expression is primarily restricted to ESC, where its suppression induces cell differentiation (5). Besides its part, as the main transcription factor, in regulating the stemness property of ESCs, OCT4 also plays a key role in modulating survival of cancer cells (6,7). *OCT4* potentially encodes at least three different variants via alternative splicing (variants A, B and B1) (8). OCT4A is exclusively expressed in the nucleus of ES and some adult stem cells, and control the pluripotency of the cells. OCT4B is widely expressed in the cytoplasm of stem and tumor cells and seems to have a role in apoptosis and stress pathways. A novel splice variant of *OCT4* (OCT4B1) has recently been reported to be expressed in both pluripotent and cancer cells by Atlasi et al. (9). Subsequent studies revealed up-regulation of OCT4B1 in gastric (8), colorectal (9), bladder (10), and germ cell tumors (11), where it functions as an anti-apoptotic factor (7,8). Moreover, it was reported by Farashahi et al. (10) that under stress conditions (heat shock), the ratio of OCT4B1/OCT4B was significantly elevated in bladder tumor cell lines. 

Heat shock proteins (HSPs) are among the most conserved proteins expressed in both prokaryotes and eukaryotes (10). A wide variety of physiological and environmental insults such as heat shock stress could alter expression of HSPs. As molecular chaperones, these proteins play fundamental roles in correct folding of nascent and stress-accumulated misfolded proteins, and hence prevent their aggregation (11). According to their location (intracellular or extracellular), HSPs play dual protective and/ or inducing functions. While intracellular HSPs help the cells to survive from lethal conditions, extracellular or membrane-bound HSPs interact with various components of the regulated programmed cell death pathways (12). In physiological conditions, HSPs are expressed in normal cells, however, under stress conditions (i.e. heat shock) their expression is up-regulated. 

Based on their molecular sizes, mammalian HSPs have been classified in two subgroups of high and small molecular weight HSPs. HSP60, HSP70 and HSP90 belong to the high molecular weight group, while HSPs with molecular weights less than 60 kDa are classified in the small molecular weight group. Moreover, some members of HSPs are constitutively expressed, whereas the expression of others is induced by stress conditions (13). Under stress conditions, HSPs could also target some selected proteins toward proteasomemediated degradation. Altogether, these properties make HSPs suitable potential targets for regulating cell death pathways (14). 

HSPs are up-regulated in a wide range of human cancers and are associated with several properties common to tumorigenesis including proliferation, invasion and metastasis (14). HSP70 gene family have anti-apoptotic potency and up-regulated in malignant human tumors. Interestingly, HSP70 gene family enhances the tumorigenic potency of rodent cells (15). 

HSP90, ATP-dependent molecular chaperones, are essential for the activation and stabilization of several proteins involved in various cellular pathways, including apoptosis. Therefore, it is widely considered as an interesting target for cancer therapy (16). 

Based on our previous finding (10) on a possible involvement of the OCT4B1 variant in stress pathways, we knocked-down the expression of OCT4B1 in three human tumor cell lines and profiled expression changes of stress-related genes in the treated cells. 

## Materials and Methods

### Cell culture

In this experimental study, AGS, 5637, and U87MG tumor cell lines were obtained from the Iranian National Cell Bank (Pasteur Institute, Iran) and cultured in RPMI-1640 (Gibco, UK) supplemented with 10% (v/v) heat-inactivated fetal bovine serum (FBS) and 2 mM L-glutamine (Sigma, USA) at 37˚C and 5% CO_2_.

### RNA extraction, reverse transcription (RT) and real-time polymerase chain reaction (PCR)

Real-time RT-PCR was carried out to evaluate the expression level of *OCT4* variants in the studied tumor cell lines. Total RNA was isolated from cultured cells (10^6^cells per milliliter), using Trizol (Invitrogen, USA) and according to the manufacturer’s guidelines. To remove any possible DNA contamination, RNA was treated with TURBO DNase. The purity and integrity of RNA were measured by spectrophotometer (260/280 nm ratio) and visual observation of the samples on agarose gel electrophoresis (1% agarose gel), respectively. The first strand of cDNA was synthesized at 42˚C for 60 minutes using 100 pmol oligo(dT) primers, 1 µg of total RNA and a cDNA synthesis kit from (ParsTous Co., Iran), according to the manufacturer’s instructions. Specific primers were designed for OCT4A, OCT4B, OCT4B1, and *ß-actin* (as a housekeeping gene) using Gene Runner (version 3.02) and Allele ID (version 4.0) softwares (Table 1). 

Quantitative real-time PCR was performed by addition of SYBR green master mix (ParsTous, Iran), 200 ng of the previously generated cDNA and 2 pg/µl of the appropriate primers. The following cycling conditions was set on a BIO-RAD CFX96 system (Bio-Rad Company, USA): one cycle of 95˚C for 15 minutes, 40 cycles of 95˚C for 30 seconds, 60˚C for 30 seconds (61˚C for 20 seconds for OCT4B1) and 72˚C for 30 seconds. Real-time PCR was carried out in triplicate and ß-actin was employed as a housekeeping gene for normalizing amplification signals of the target genes. The relative amounts of PCR products were determined using the 2 ^-∆∆Ct^ formula. The dissociation stages, melting curves and quantitative analyses of the data were performed using the CFX manager software version 1.1.308.111 (Bio-Rad, USA). All PCR products were visualized with agarose gel electrophoresis to confirm the uniqueness and correct size of the PCR products. 

**Table 1:** Sequences of the primers used for siRNA and real-time PCR


Name	Direction	Sequences

VersionI OCT4B1:siRNA	Target	AAGGAGTATCCCTGAACCTAG
	Sense	(GGAGUAUCCCUGAACCUAG)dTdT
	Anti-sense	(CUAGGUUCAGGGAUACUCC)dTdT
VersionII OCT4B1:siRNA	Target	AAGAGGTGGTAAGCTTGGATC
	Sense	(CAGUGGUAAGCUUGGAUC)dTdT
	Anti-sense	(AAUCCAAGCUUACCACCUC)dTdT
Scramble:siRNA	Sense	(GCGGAGAGGCUUAGGUGUA)dTdT
	Anti-sense	(UACACCUAAGCCUCUCCGC)dTdT
OCT4A primer	F	CGCAAGCCCTCATTTCAC
	R	CATCACCTCCACCACCTG
OCT4B primer	F	CAGGGAATGGGTGAATGAC
	R	AGGCAGAAGACTTGTAAGAAC
OCT4B1 primer	F	GGTTCTATTTGGTGGGTTCC
	R	TTCTCCCTCTCCCTACTCCTC
ß-actin primer	F	CACACCTTCTACAATGAGC
	R	ATAGCACAGCCTGGATAG


PCR; Polymerase chain reaction.

### OCT4B1 siRNA and scramble siRNA transfection

In order to suppress OCT4B1, two specific siRNAs complementary to the unique region of theOCT4B1 sequence (exon2b), and one irrelevant/ scrambled siRNA (with no complementary target sequence in the human genome, as control) were designed, using the siRNA selection program (Whitehead Institute for Biomedical Research, http://jura.wi.mit.edu/, MWG Co., Germany) (Table 1). 

Tumor cell lines (1×10^5^ cells/ml) were cultured in six-well plates in RPMI-1640 medium without antibiotics in two groups (test and control). At a confluency of 30-50%, cells were transfected with 50 nmol/ml of OCT4B1-siRNA (scramble siRNA for the control group) using lipofectamin 2000 (Invitrogen, USA) and opti-MEM media according to the manufacturer’s instructions. Briefly, 5 μl of siRNA (25 μM) and 4.5 μl RNAi-MAX were diluted in 250 μl Opti-MEM and incubated for 10 minutes at room temperature. The mixture was then added to the cells in a final volume of 2.5 ml, and incubated at 37˚C in a humidified atmosphere and 5% CO_2_ for 72 hours.

To determine the efficiency of gene suppression, OCT4B1 expression was quantified in OCT4B1siRNA (test group) and scramble-siRNA (control group) transfected cells at 24, 48 and 72 hours after cell transfection. 

### High molecular weight heat shock proteins genes profiling

Forty-eight hours after transfection, cells were heat-shock treated at 45˚C for 1 hour (10). Total RNA was extracted from cells of the test and control groups, and cDNA was synthesized immediately as described previously (17). Quantitative gene expression analysis was performed using a real-time PCR array approach (SABiosciences Company, USA). 

### Statistical analysis

Gene expression analysis and chart drawing were carried out using the CFX96 manager software (Bio-Rad, USA) and RT2 Profiler PCR Array Data Analysis (version 3.5) respectively. The statistical analysis was carried out by one-way ANOVA (SPSS software, version 18). 

## Results

### OCT4 variants were expressed in all examined cell lines and down-regulated after siRNA transfection

**Fig.1:**
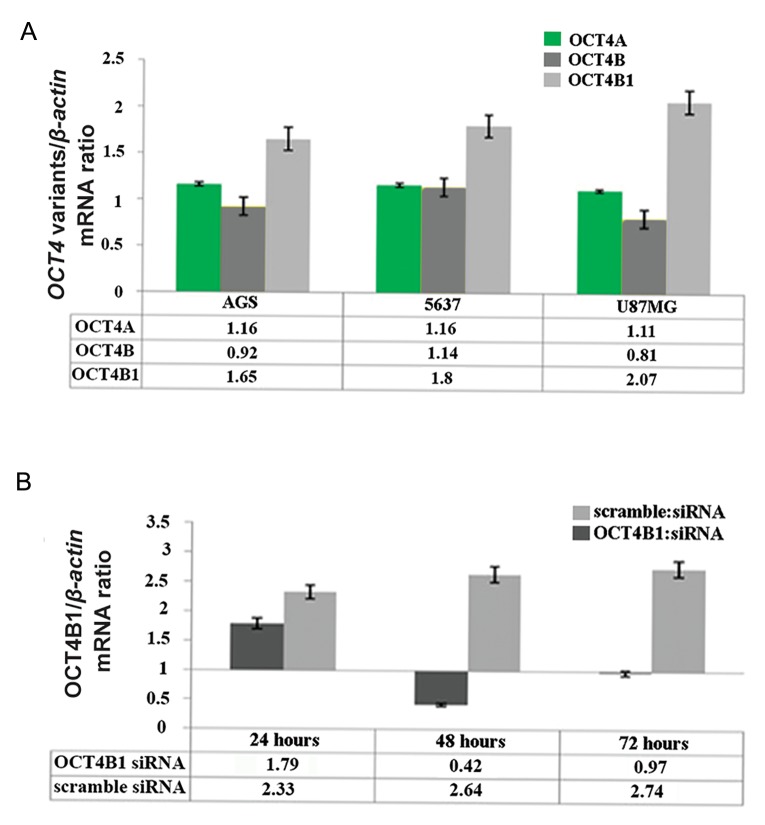
A. The expression levels of OCT4 variants in U87MG, 5637, and AGS cell lines. Y axis shows OCT4 variants mRNA expression level normalized to that of ß-actin (as a housekeeping control gene) and B. OCT4B1 mRNA expression level at 24, 48 and 72 hours after transfecting AGS cells with OCT4B1 or scramble siRNAs. OCT4B1 expression level is dramatically reduced 48 hours after transfection with OCT4B1 siRNA, confirming the specificity of the designed siRNA.

Our real-time PCR data demonstrated that all three OCT4 variants were expressed in all three cell lines (Fig .1A), with the highest expression level of OCT4B1 observed in the U87-MG cell line. As expected, after siRNA transfection, OCT4B1 expression was dramatically decreased at 24, 48 and 72 hours. As shown in figure 1B, the highest level of OCT4B1 suppression was observed at 48 hours post-transfection. 

### Expression alteration of HSP60, HSP70 and HSP90 gene families following OCT4B1 suppression

**Fig.2:**
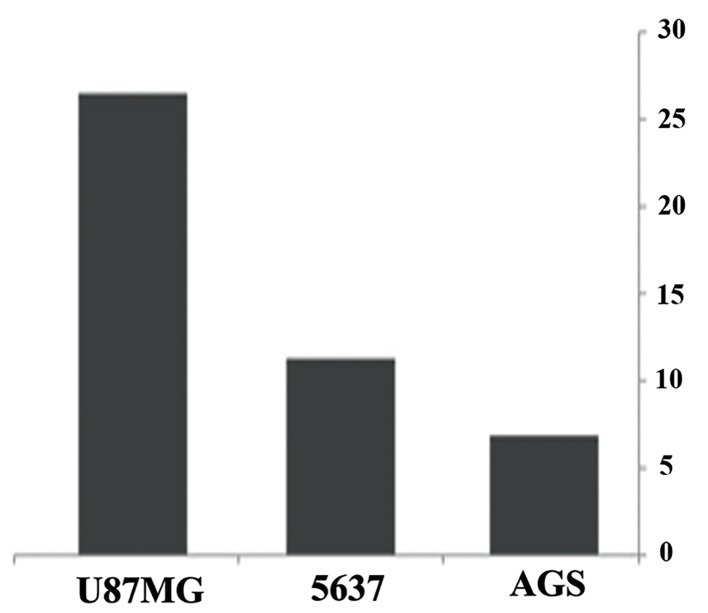
Altered expression of *HSPD1* (member of the HSP60 gene family) at 48 hours after OCT4B1: siRNA transfection in U87MG, 5637 and AGS cell lines. Y axis shows the fold changes in gene expression.

**Fig.3:**
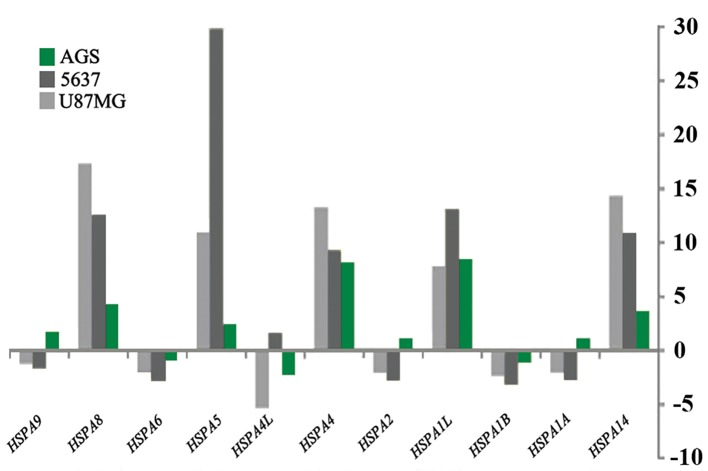
Altered expression of HSP70 gene family members at 48 hours after OCT4B1:siRNA transfection in U87MG, 5637 and AGS cell lines. Y axis shows the fold changes in gene expression.

**Fig.4:**
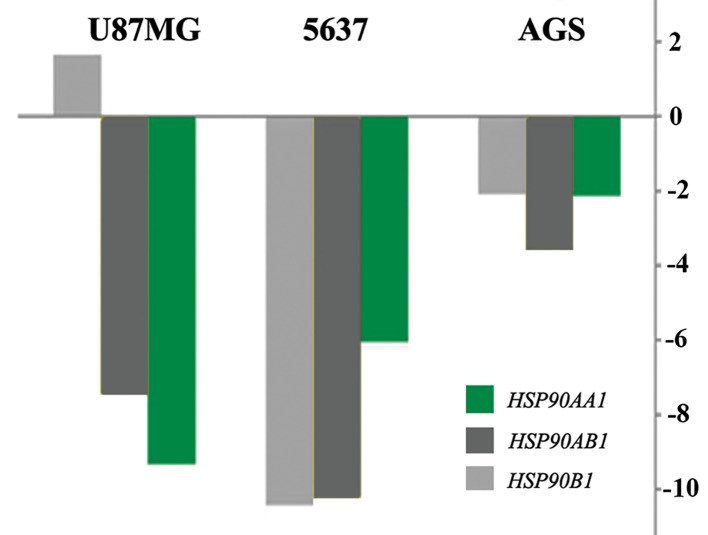
Altered expression of HSP90 gene family members, 48 hours after OCT4B1:siRNA transfection in U87MG, 5637 and AGS cell lines. Y axis shows the fold changes in gene expression.

Results of real-time PCR revealed an similar but not identical pattern of expression alteration in all three studied tumor cell lines. For instance, *HSPD1*, a member of the HSP60 gene family, was upregulated in all three tumor cell lines, following OCT4B1 suppression (Table 2,Fig.2). Five out of eleven members of the HSP70 gene family were up-regulated, whereas two genes were down-regulated after OCT4B1 silencing. Although *HSPA1A*, *HSPA2*, and *HSPA9* genes showed down-regulation in AGS and 5637 cells, they were up-regulated in U87MG cells. Similar inconsistency was observed for *HSPA4L* which showed down-regulation in AGS and U87MG cells and up-regulation in 5637 cells (Table 2,Fig.3). Three members of the HSP90 gene family (*HSP90AA1*, *HSP90AB1*, and *HSP90B1*) analyzed were all down-regulated following OCT4B1 suppression in all three tumor cell lines except for *HSP90B1* in AGS cells that showed less than 2-fold up-regulation (Table 1,Fig .4). 

**Table 2:** Altered expressions of HSP60, HSP70, and HSP90 gene families in AGS, 5637 and U87MG tumor cell lines at 48 hours after OCT4B1 suppression


Gene categories/name	Description of genes	Fold cAGS	hanges/c5637	ell linesU87MG

HSP90 family members				
*HSP90AA1*	Heat shock protein 90 kDa alpha (cytosolic), class A member 1	-9.32	-6.04	-2.12
*HSP90AB1*	Heat shock protein 90 kDa alpha (cytosolic), class B member 1	-7.48	-10.25	-3.60
*HSP90B1*	Heat shock protein 90 kDa beta (Grp94), member 1	1.65	-10.42	-2.08
HSP70 family members				
*HSPA14*	Heat shock 70 kDa protein 14	14.31	10.89	3.62
*HSPA1A*	Heat shock 70 kDa protein 1A	-2.05	-2.80	1.02
*HSPA1B*	Heat shock 70 kDa protein 1B	-2.35	-3.22	-1.13
*HSPA1L*	Heat shock 70 kDa protein 1-like	7.87	13.10	8.48
*HSPA2*	Heat shock 70 kDa protein 2	-2.08	-2.84	1.00
*HSPA4*	Heat shock 70 kDa protein 4	13.26	9.29	8.10
*HSPA4L*	Heat shock 70 kDa protein 4-like	-5.33	1.62	-2.18
*HSPA5*	Heat shock 70 kDa protein 5 (glucose-regulated protein, 78 kDa)	11.02	29.83	2.35
*HSPA6*	Heat shock 70 kDa protein 6	-2.11	-2.88	-1.01
*HSPA8*	Heat shock 70 kDa protein 8	17.30	12.63	4.38
*HSPA9*	Heat shock 70 kDa protein 9 (mortalin)	-1.20	-1.64	1.73
HSP60 family member				
*HSPD1*	Heat shock 60 kDa protein 1 (chaperonin)	26.46	11.25	6.84


PCR; Polymerase chain reaction.

## Discussion

In our previous work (10), we presented evidence suggesting a potential role for OCT4B1 in stress response. Due to the direct link of stress response in cancer initiation, we aimed here to decipher the molecular targets in stress pathways regulated by OCT4B1. For that reason, we examined expression changes of high molecular weight HSPs (HSP60, HSP70 and HSP90) following OCT4B1 suppression in three different tumor cell lines. 

In contrast to genes in the HSP70 gene family, the three members of the HSP90 gene family (*HSP90AA1*, *HSP90AB1*, and *HSP90B1*) were slightly down-regulated, suggesting that this gene family is a target of OCT4B1 mediated stress pathway. *HSPD1*, the candidate gene from the HSP60 gene family, showed up-regulation in all three examined cell lines. 

Our data revealed some cell-type specific effects among the three cell lines. For example, while *HSPA1A*, *HSPA2*, and *HSPA9*, the members of HSP70 gene family, were down-regulated in AGS and 5637 cells, they showed upregulation in U87MG cells. Similarly, *HSPA4L* was down-regulated in AGS and U87MG cells, while up-regulated in 5637 tumor cell line. 

It has been evidenced that the members of the HSP90 gene family are essential for cell cycle regulation, survival, and apoptosis (18). These molecules primarily participate in maintenance of cellular protein homeostasis, by acting as molecular chaperones to ensure correct folding of nascent proteins. Interestingly, the HSP90 gene family is considered as a novel therapeutic target for can certherapy (18). Heat, reactive oxygen species, *γ-irradiation* and injury are the main inducers of *HSP90* expression by tumor cells (19). Members of this gene family interact with over 400 client proteins, indicating their importance in cell behavior regulation. The client molecules are involved in mediating signal transduction pathways which lead to cellular growth and apoptotic evasion (20). Our results demonstrated that following OCT4B1 suppression, the expression level of *HSP90* genes were decreased, suggesting that up-regulation of OCT4B1 in cancer cells and tissues induces the expression of *HSP90* genes. Centenera et al. (21) reported that *HSP90* proteins play significant roles in the stability of critical signaling proteins important in initiation and progression of prostate cancer. Moreover, Niknejad et al. (22), reported that *HSP90* suppress apoptosis, enhances angiogenesis and promote cell cycle progression in cancer cells. Accordingly, upregulation of *HSP90* genes has been reported in cancer tissue samples (23). 

Over-expression of OCT4B1 in cancer cells and tissues (22), it santi-apoptotic effect (24) and its association with stress signaling pathways (25) imply that OCT4B1 up-regulation in tumor cells could lead to over-expression of HSP90 members. Accordingly, this association may be considered as a probable mechanism for mediating the anti-apoptotic role of OCT4B1 in tumor cells. In other words, down-regulation of OCT4B1 could be considered as an alternative strategy for HSP90-mediating molecular therapy of cancers. 

The HSP70 family is mostly considered as a potent buffering system for cellular stress which is necessary for cancer cell survival (26). Therefore, it has been proposed that the HSP70 family serve as crucial survival mediators in the cells. Several reports exist on the critical role of HSP70 molecules in cancer progression (26,28). However, there is little knowledge on the molecular mechanisms by which HSPs mediate survival. According to our results, following OCT4B1 suppression in studied tumor cell lines, the expression level of 5 out of 11 members of the HSP70 gene family was increased, while, two genes showed down-regulation and four remaining members showed no significant alteration in their expression level. Thus, we failed to demonstrate direct co-expression of OCT4B1 and HSP70 genes. 

Significant changes in HSP60 expression during the progression of some tumors suggest that these molecules play a critical role in carcinogenesis. HSP60 is mainly expressed in mitochondria, however their expression has been also reported within cytosol, on the cell membrane as well as in intracellular vesicles and extracellular spaces (29). Interestingly, it has been reported that HSP60 are up-regulated in medulloblastoma (30), colorectal cancer (31), leukemia (32), breast carcinoma (33) and prostate cancer (34). Hence, it appears that HSP60 expression may be associated with enhanced proliferation of cancer cells. The overexpression of *HSPD1*, a member of HSP60 family, following OCT4B1 suppression led us to hypothesize that up-regulation of HSPD1 is a normal response of tumor cells to escape from apoptosis and that it may not be a direct target of OCT4B1. Accordingly, it seems that the intracellular regulation of HSPs in tumors is complex, and also cell-type specific. 

## Conclusion

Our data support our previous report on a possible direct association between the expression of OCT4B1 and the HSP90 gene family. Accordingly, suppression of OCT4B1 in different tumor cell lines caused significant down-regulation of the members of the HSP90 family. However, a direct link between the expression level of OCT4B1 and those of HSP70 and HSP60 gene families were less evident. 
